# Magnetorheological Response for Magnetic Elastomers Containing Carbonyl Iron Particles Coated with Poly(methyl methacrylate)

**DOI:** 10.3390/polym13030335

**Published:** 2021-01-21

**Authors:** Daichi Takahashi, Annadanam Venkata Sesha Sainath, Junko Ikeda, Kulisara Budpud, Tatsuo Kaneko, Mika Kawai, Tetsu Mitsumata

**Affiliations:** 1 Graduate School of Science and Technology, Niigata University, Niigata 950-2181, Japan; f19b020g@mail.cc.niigata-u.ac.jp (D.T.); f17k501b@mail.cc.niigata-u.ac.jp (J.I.); mikagoro@eng.niigata-u.ac.jp (M.K.); 2 Fluoro-Agrochemicals, Polymers and Functional Materials Department, CSIR-Indian Institute of Chemical Technology, Hyderabad 500007, India; avss@iict.res.in; 3 Graduate School of Advanced Science and Technology, Japan Advanced Institute of Science and Technology, Nomi 923-1292, Japan; b.kulisara@jaist.ac.jp (K.B.); kaneko@jaist.ac.jp (T.K.)

**Keywords:** soft material, stimuli-responsive gel, magnetic elastomer

## Abstract

The magnetorheological response for magnetic elastomers containing carbonyl iron (CI) particles with a diameter of 6.7 μm coated with poly(methyl methacrylate) (PMMA) was investigated to estimate the diameter of secondary particles from the amplitude of magnetorheological response. Fourier-transformed infrared spectroscopy revealed that the CI particles were coated with PMMA, and the thickness of the PMMA layer was determined to be 71 nm by density measurement. The change in the storage modulus for magnetic elastomers decreased by coating and it was scaled by the number density of CI particles as Δ*G*~*N*^2.8^. The diameter of secondary particle of CI particles coated with PMMA was calculated to be 8.4 μm. SEM images revealed that the CI particles coated with PMMA aggregated in the polyurethane matrix.

## 1. Introduction

Magnetic elastomers are stimuli-responsive, soft materials [1,2,3,4,5], and their physical properties alter in response to magnetic fields. The magnetic response for a magnetic elastomer is in general drastic; therefore, the materials attract considerable attention as actuators in the next generation [6,7,8,9,10]. A magnetic elastomer consists of polymeric matrices such as polyurethane, and magnetic particles nanometers or micrometers in diameter. When a magnetic field is applied to magnetic elastomer, the elasticity increases due to the chain structure formation (restructuring) of magnetic particles, which is called the magnetorheological (MR) effect. We have investigated so far the methodology for an efficient MR effect by weak magnetic field to use magnetic soft materials practically [11,12,13,14,15].

In general, it is necessary to obtain an efficient MR effect such that magnetic particles are highly dispersed in the polymer matrix [16,17,18,19]. That is because that the MR amplitude is reduced by the formation of secondary particles of magnetic particles. Hence, avoiding the occurrence of secondary particles leads to an increase in the MR amplitude. There is a variety of methods to determine the diameter of secondary particles in the polymeric matrix—e.g., optical microscopy, confocal laser microscopy, particle-size analysis, etc. However, only few methods, such as X-ray CT and particle size analysis by ultrasound, can be used for magnetic elastomers, since they are not transparent optically. Ikeda et al. proposed a simple method to determine the diameter of secondary particles of magnetic particles in magnetic elastomers [20]. The change in storage modulus exhibited a power dependency against the number of magnetic particles, which was nearly independent of the magnetic particles. The change in storage modulus was successfully scaled by the reduced number of magnetic particles using the diameter of secondary particles. Microphotographs revealed that the diameter of secondary particles was similar to that determined from the reduced number of magnetic particles.

In this study, the relationship between the change in storage modulus and the reduced number of magnetic particles was examined using carbonyl iron particles with and without a coating of poly(methyl metacrylate) (PMMA). Through the coating, the chemical affinity between carbonyl iron particles and polyurethane matrix is changed. Choi et al. has been widely and systematically investigating the effect of coating on the magnetic response for magnetic soft materials, e.g., PMMA/carbonyl iron and PS/carbonyl iron [21,22,23,24,25]. It was found that the synthesized core–shell structured CI particles possess better dispersion stability in suspending oil than bare CI particle suspension, due to the density reduction of the particles [22]. This report tells us that the dispersibility of CI particles coated with PMMA in a matrix of polyurethane decreases compared to bare CI particles. Since the coating layer is very thin, one can easily find the differences in the particle dispersibility and MR response for these particles. Motivated by this, we prepared magnetic elastomers embedded with CI particles having thin layers of PMMA and investigated the effect of coating on the magnetorheological response.

## 2. Experimental procedures

### 2.1. Synthesis of Magnetic Elastomers and Gels

Polypropylene glycols (P2000, G3000B, Adeka Co., Tokyo, Japan) with molecular weights of *M*_w_ = 2000 and 3000 were used for the matrix of magnetic elastomers. Tolyrene diisocyanate (Wako Pure Chemical Industries. Ltd., Osaka, Japan) and dioctyl phthalate (DOP, Wako Pure Chemical Industries. Ltd., Osaka, Japan) were used for a crosslinker and plasticizer, respectively. Carbonyl iron with a median diameter of 6.7 μm (CS Grade BASF SE., Ludwigshafen am Rhein, Germany) was used for magnetic particles. The saturation magnetization of CI particles was 190 emu/g measured by SQUID magnetometer (MPMS, Quantum Design Inc., San Diego, CA, USA).

The PMMA (Wako Pure Chemical Industries. Ltd., Osaka, Japan) coating on the CI surface was carried out by the following procedure. A 10 wt% of PMMA solution was prepared by dissolving PMMA in tetrahydrofuran (THF). CI particles were dispersed in THF (Wako Pure Chemical Industries. Ltd., Osaka, Japan); then the dispersion was poured in the PMMA solution with stirring. The powder of PMMA/CI was obtained by vacuum filtration and the residue was dried at 50 °C for 12 hours.

Magnetic elastomers were synthesized using a prepolymer method. Polypropylene glycols were crosslinked with tolyrene diisocyanate. The molar ratio of –NCO to –OH group for the prepolymer was constant at 2.01 (=[NCO]/[OH]). CI particles were mixed with prepolymer, linear polymer, plasticizer and catalysis. The mixed liquid was poured into a silicon mold and cured for 30 min at 100 °C. The weight concentration of CI particles was varied up to 85 wt%, and the weight concentration of DOP to the matrix without CI particles was fixed at 60 wt%. The density of CI particles was 7.57 g/cm^3^.

A pre-gel solution of the magnetic hydrogel was prepared by mixing 1 wt% carrageenan (*M*_w_ = 857 kDa, CS-530, San-Ei Gen F.F.I., Osaka, Japan) aqueous solution and magnetic particles at 100 °C using a vortex mixer for approximately 1 min. The weight concentration of magnetic particles was kept at 70 wt%.

### 2.2. Dynamic Viscoelastic Measurement

Dynamic viscoelastic measurement was carried out for magnetic elastomers using a rheometer (MCR301, Anton Paar Pty. Ltd., Graz, Austria) at 20 °C. The strain was varied from 10^−5^ to 1 and the frequency was kept at 1 Hz. The sample was a disk 20 mm in diameter and 1.5 mm in thickness. The normal force initially applied to the magnetic elastomer was approximately 0.3 N.

### 2.3. SEM Observation

**Figure 1:**
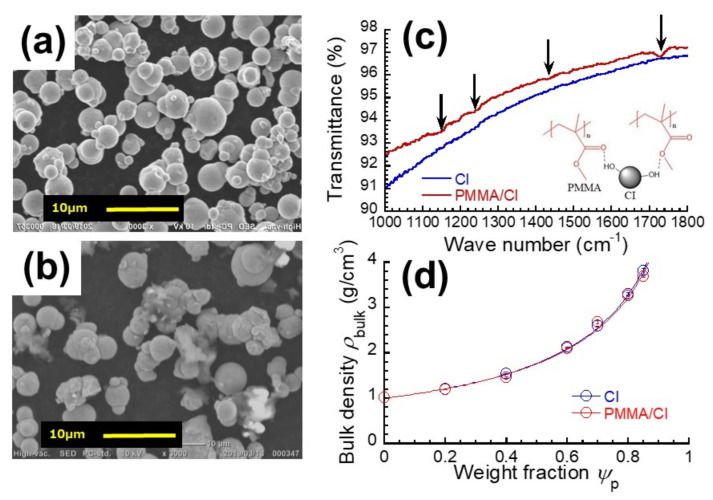
SEM photographs for CI particles (**a**) without and (**b**) with PMMA coating. (**c**) FT-IR spectra for CI particles without and with PMMA coating. (**d**) Bulk density of magnetic elastomers containing CI and PMMA/CI particles with various volume fractions of CI particles.

The shape of CI particles coated with PMMA and the dispersibility of CI particles in the magnetic elastomer were observed using scanning electron microscopy (SEM, JCM-6000 Neoscope JEOL Ltd. Tokyo, Japan) with an accelerating voltage of 15 kV without Au coating. SEM photographs for CI particles with and without coatings are presented in Figure 1a,b, respectively.

### 2.4. FT-IR Spectroscopy

Fourier-transformed infrared (FT-IR) spectra for CI particles with and without coatings were measured in a wavenumber range of 600–4000 cm^−1^ at room temperature, using a FT-IR spectrometer (Spectrum One spectrometer, Perkin-Elmer, Waltham, MA, USA) with diamond attenuated total reflection (ATR) accessories. The IR spectra are presented in Figure 1c.

### 2.5. Thermogravimetric Analysis

Thermogravimetric analysis (TGA) was performed on a thermogravimetric analyzer (STA 7200, Hitachi Hi-Tech. Sci. Co., Tokyo, Japan). The samples were preheated to eliminate the moisture at 100 °C for 1 h before TGA. TGA was measured under a nitrogen atmosphere in the temperature range of 25–500 °C, and the heating rate was set to 10 °C/min with a 10 min holding time.

## 3. Results and Discussion

Figure 1d exhibits the relationships between bulk density and the weight fraction for CI particles with and without coatings. The bulk density ρ_bulk_ increased with the weight fraction of CI particles satisfying the following equation,

(1) $$\frac{1}{\rho_{\mathrm{bulk}}}=\frac{\emptyset_{p}}{\rho_{p}}+\frac{1-\emptyset_{p}}{\rho_{m}}$$

where the *ϕ*_p_ represents the volume fraction of CI particles and the *ρ*_p_ and *ρ*_m_ are the densities of polymer matrix and CI particles, respectively. The experimental data of *ρ*_bulk_ were fitted by the above equation using a value of *ρ*_p_ = 1.060 g/cm^3^. The values of *ρ*_m_ before and after coating were determined to be 7.700 and 7.325 g/cm^3^, respectively. The volume fraction of PMMA *ϕ*_PMMA_ for the CI particle with PMMA coating was calculated to be 0.0576 using a value of *ρ*_PMMA_ = 1.188 g/cm^3^. The result of TGA revealed that a decrease in weight of 0.9898% was observed at 450 °C for PMMA/CI particles; meanwhile, no weight change was observed for CI particles at temperatures below 500.0 °C. The *ϕ*_PMMA_ for the CI particles with PMMA coatings was calculated to be 0.060, which coincided with the data obtained from the density measurement. The thickness of PMMA layer surrounding a CI particle δ*r* can be estimated by the following equation.

(2) $$\phi_{\mathrm{PMMA}}=1-{\left(\frac{r}{r+\delta r}\right)}^{3}$$

where *ϕ*_PMMA_ is the volume fraction of PMMA and *r* is the radius of primary particle of CI (= 3.35 μm). The thickness of PMMA layer was calculated to be 71 nm, which corresponds to 1.1% of the diameter of primary particle. Figure 1a,b shows the SEM photographs for CI particles without and with PMMA coatings. Figure 1b reveals that the CI particle was coated with PMMA while keeping its spherical shape. Figure 1c exhibits the IR spectra for CI particles without and with coatings. The spectrum with coating showed the clear peaks related to the ester groups of PMMA at wavenumbers of 1148 (C–O–C), 1236 (C–C–O) and 1731 (C=O stretching) cm^−1^; and a peak of main-chain alkyls at 1432 cm^−1^. The peaks were relatively weak; however, the spectra strongly indicate that PMMA was coated on CI particles by self-adhesion. Similarly to the previous literature [26], it can be considered that hydrogen bonding occurs between PMMA ester groups of –O– and C=O with CI peripheral OH groups, as schematically illustrated in the inset of Figure 1c.

**Figure 2:**
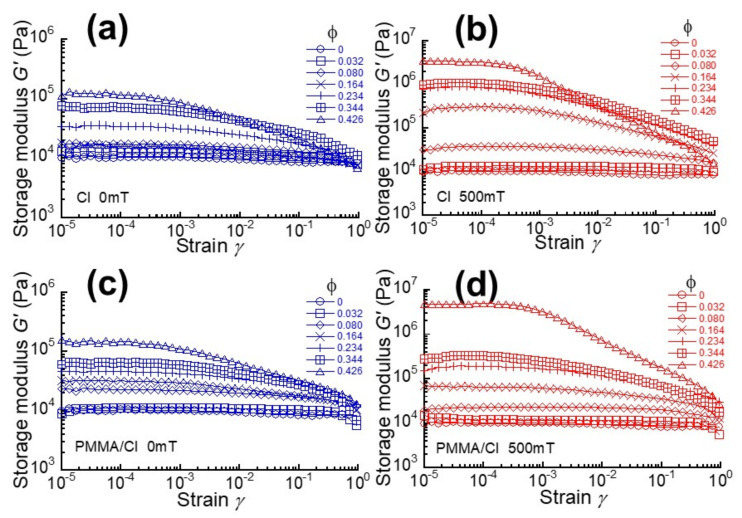
Strain dependence of storage modulus at (**a**, **c**) 0 mT and (**b**, **d**) 500 mT for magnetic elastomers containing CI particles without and with PMMA coatings: (**a**, **b**) CI, (**c**, **d**) PMMA/CI.

Figure 2a–d demonstrate the strain dependence of storage modulus at 0 mT and 500 mT for magnetic elastomers without and with coatings. At 0 mT, the storage modulus for magnetic elastomers was almost independent of the strain, suggesting that an apparent particle network of CI particles is not formed in the elastomer, which coincides with our previous studies [16,17]. At 500 mT, the storage modulus at low strains was increased by the magnetic field, which is a response typically observed for magnetorheological elastomers or gels. At high strains, the increase in the storage modulus was also seen; however, the increment in the modulus was small compared to that at low strains, which is also typical behavior of magnetorheological elastomers. It is worth mentioning that the linear viscoelastic regime for magnetic elastomers with PMMA/CI was extended to high strains. For example, the onset strain of nonlinear viscoelasticity at 500 mT for magnetic elastomers with ϕ = 0.426 was approximately 5 × 10^−4^ for PMMA/CI and 2 × 10^−4^ for CI particles, respectively. This strongly indicates that the diameter of CI particle increased by the coating of PMMA.

**Figure 3:**
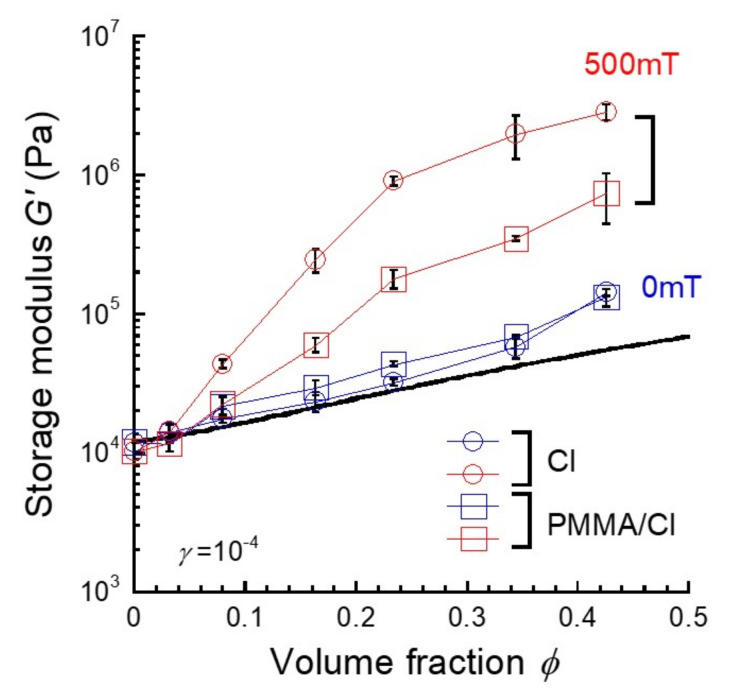
Relationship between storage modulus and the volume fraction of CI particles for magnetic elastomers containing CI particles without and with PMMA coatings. The solid line represents the Guth–Gold equation.

Figure 3 depicts the storage modulus for magnetic elastomers as a function of the volume fraction of magnetic particles for magnetic elastomers with and without PMMA coatings. The storage modulus at 0 mT for magnetic elastomers without coatings obeyed the following Guth–Gold equation [27]:

(3) $$G^{’}=G_{0}^{’}\left(1+2.5\phi +14.1\phi^{2}\right)$$

where *G*_0_ and *ϕ* are the storage modulus for a polyurethane elastomer without CI particles and the volume fraction of CI particles, respectively. The storage modulus for magnetic elastomers with coatings was slightly higher than that for magnetic elastomers without coatings, suggesting the occurrence of the aggregation of CI particles. At 500 mT, the storage modulus for magnetic elastomers without coatings exceeded 1 MPa, indicating that CI particles form a well-developed chain structure in the polyurethane network, as in [28,29,30]. Meanwhile, for magnetic elastomers with coatings, it significantly leveled off (1/2 of without coating). This strongly indicates that the number of chains of CI particles decreased by the coating of PMMA.

**Figure 4:**
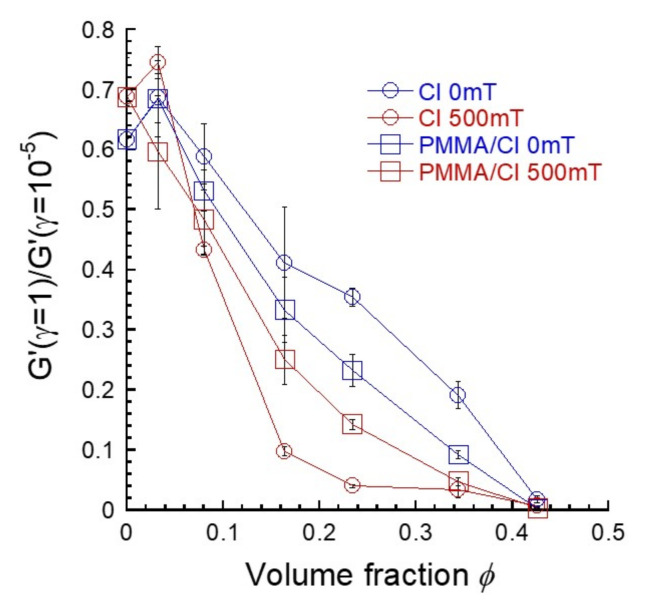
Relationship between the ratios between storage moduli at high and low strains G’(γ = 1)/G’(γ = 10^−5^) for magnetic elastomers and the volume fractions of CI particles without and with PMMA coatings.

Figure 4 exhibits the relationship between the amplitude of Payne effect and the volume fraction of CI particles for magnetic elastomers without and with PMMA coatings. The ratio of storage moduli at high and low strains *G*’(*γ* = 1)/*G*’(*γ* = 10^−5^), which shows the amplitude of Payne effect, was calculated using the values of storage moduli at strains of *γ* = 1 and 10^−5^. At 0 mT, the values of the ratio for CI particles with coatings were similar to those with coatings at *ϕ* < 0.07, suggesting no clear difference in the particle dispersibility for these particles. Meanwhile, at *ϕ* > 0.17, the values of the ratio for CI particles with coatings were clearly lower than those without coatings, indicating the occurrence of the secondary particles of CI. Similarly, at 500 mT, the values of the ratio for CI particles without coatings were lower than those with coatings at *ϕ* > 0.17. This strongly indicates that CI particles without coatings have many contacts between the particles in the chain structure, compared to CI particles with coatings.

**Figure 5:**
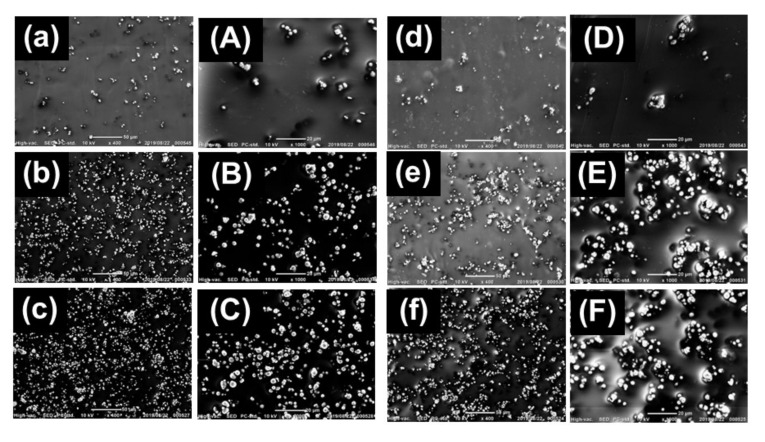
SEM photographs for magnetic elastomers containing CI particles (**a**–**c**, **A**–**C**) without and (**d**–**f**, **D**–**F**) with PMMA coatings. Magnification (**a**–**f**): ×400, (**A**–**F**): ×1000.

Figure 5 shows the SEM photographs for magnetic elastomers containing CI particles without and with PMMA coatings. CI particles without coatings were randomly dispersed in a matrix of polyurethane as primary particles, which agrees with our previous reports [16,17]. Most of these photographs showed that CI particles coated with PMMA aggregated and formed secondary particles in the matrix.

**Figure 6:**
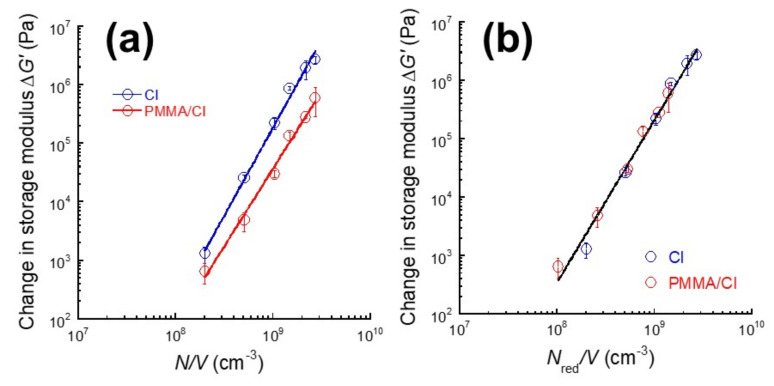
Changes in storage modulus due to magnetic field as functions of (**a**) number density of CI particles and (**b**) reduced number density of CI particles for magnetic elastomers containing CI particles without and with PMMA coatings.

Figure 6a demonstrates the relationship between the change in storage modulus and the number density of CI particles for magnetic elastomers without and with PMMA coatings. The number density of CI particles divided by the sample volume *N/V* was calculated from the following equation.

(4) $$\frac{N}{V}=\frac{\emptyset }{\frac{4}{3}\pi {\left(\frac{D_{p}}{2}\right)}^{3}}$$

where *ϕ* is the volume fraction of CI particles and *D*_p_ is the diameter of primary particle (= 6.7 μm). The slopes for CI particles without and with PMMA coatings were similar, indicating that the Δ*G’* can be scaled by the number density of CI particles.

Similarly to our previous study [20], the diameter of secondary particles was determined using the reduced number density of CI particles. Figure 6b demonstrates the relationship between the change in storage modulus and the reduced number density of CI particles for magnetic elastomers without and with PMMA coatings. The dominant parameter of the amplitude of MR effect is the reduced number density of CI particles. The reduced number density of CI particles was calculated from Equation (4) using the diameter of secondary particles *D*_s_ instead of *D*_p_. The Δ*G’* for CI particles with and without coatings was successfully scaled as Δ*G’*~*N_red_*^2.8^ with a correlation coefficient of 0.980, which is in good agreement with our previous analysis [20]. We hypothesized that the diameter of CI particle equals the median diameter of primary particle (= 6.7 μm), since the SEM photographs showed high dispersibility of CI particles. Thus, we obtained the diameter of secondary particles of CI with coatings as 8.4 μm. The exponent on *N_red_* for magnetic hydrogels consisting of carrageenan and CI particles was 3.2, which is close to the value obtained here.

**Figure 7:**
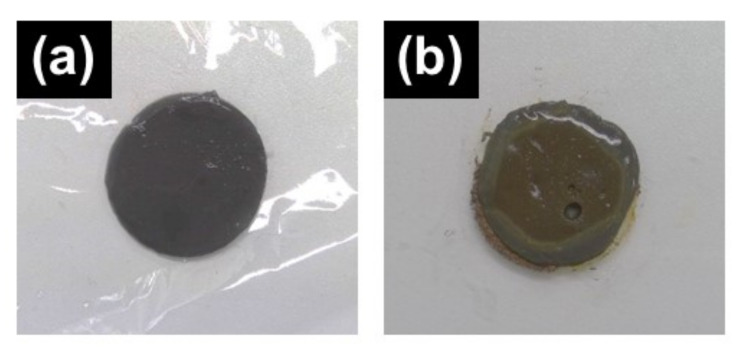
Photographs showing the time-deterioration of CI particles (**a**) with and (**b**) without PMMA coatings for magnetic hydrogels stored in a room temperature for 2 weeks.

Figure 7 demonstrates the photographs representing the time-deterioration of magnetic hydrogels containing CI particles with and without PMMA coatings. As seen in Figure 7a, the appearance of the magnetic hydrogel containing PMMA/CI particles was the same as it was immediately after the synthesis, indicating no clear deterioration occurred two weeks after the synthesis. In Figure 7b, rust due to the oxidation of CI particles can be clearly observed. It can be considered that the surfaces of CI particles were uniformly coated by PMMA, as described in Figure 1. 

## 4. Conclusions

The effect of PMMA coating on the magnetorheological response for magnetic elastomers was investigated. The change in the storage modulus due to a magnetic field decreased thanks to the coating, and it was scaled by the reduced number density of magnetic particles as ΔG~*N_red_*^2.8^. The diameter of secondary particles of carbonyl iron coated with PMMA was calculated to be 8.4 μm, which is larger than that of primary particle (= 6.7 μm). SEM images, the over-deviation from Guth–Gold formula and the amplitude of Payne effect revealed that the magnetic particles coated with PMMA aggregated in the polyurethane matrix. We believe that this method would be useful to estimate the diameters of secondary particles in magnetic elastomers.

## Data Availability

Please refer to suggested Data Availability Statements in section “MDPI Research Data Policies” at https://www.mdpi.com/ethics.
